# Association between Cardiovascular Health, C-Reactive Protein, and Comorbidities in Spanish Urban-Dwelling Overweight/Obese Hypertensive Patients

**DOI:** 10.3390/jcdd10070300

**Published:** 2023-07-13

**Authors:** Ana María Armas-Padrón, Miriam Sicilia-Sosvilla, Pedro Ruiz-Esteban, Armando Torres, Domingo Hernández

**Affiliations:** 1 La Cuesta Primary Healthcare Centre, Universidad de la Laguna, La Laguna, E-38320 Tenerife, Spain; amarmpad@gmail.com (A.M.A.-P.); mirsicsos@hotmail.com (M.S.-S.); 2 Nephrology Department, Hospital Regional Universitario de Málaga, University of Málaga, Instituto de Investigación Biomédica de Málaga (IBIMA) Plataforma BIONAND, REDinREN (RD16/0009/0006) and RICORS2040 (RD21/0005/0012), E-29010 Málaga, Spain; pedro_ruiz_esteban@hotmail.com; 3 Nephrology Department, Instituto de Tecnologías Biomédicas-Universidad La Laguna, Hospital Universitario de Canarias, REDinREN (RD16/0009/0031), E-38320 Tenerife, Spain; atorresram@gmail.com

**Keywords:** hypertension, overweight, obesity, low-grade inflammation, serum high-sensitivity C-reactive protein, hypertension-mediated organ damage, hypertension-related comorbidities, cardiovascular health metrics, cardiovascular risk factors, mortality

## Abstract

The relationship between poorer cardiovascular health metrics (CVHM) plus low-grade inflammation (LGI) and hypertension-mediated organ damage (HMOD) and hypertension-related comorbidities (HRC) in hypertensive populations with an overweight/obese (Ow/Ob) hypertension-related phenotype is understudied. We examined the relationship between the CVHM score and the presence of LGI and Ow/Ob hypertension-associated phenotype morbidities and mortality in 243 hypertensive patients from an urban primary care center. We recorded the baseline CVHM score plus clinical data, including hs-C-reactive protein (CRP) and prevalent and incident HMOD-HRC and death. A total of 26 (10.7%) had a body mass index (BMI) < 25 kg/m^2^, 95 (31.1%) were overweight (BMI 25–29.9 kg/m^2^), and 122 (50.2%) were obese (BMI ≥ 30 kg/m^2^). There were 264 cases of HMOD-HRC and 9 deaths. Higher hs-CRP levels were observed as BMI increased. Linear regression analysis showed a significant correlation between BMI and hs-CRP, adjusted for confounders. Additionally, individuals with a higher hs-CRP tertile had a significant increase in BMI. Significantly lower log hs-CRP levels were found as the number of ideal CVHM scores rose. Multivariate binary logistic regression found the risk of HMOD-HRC increased significantly as the ideal CVHM scores decreased, and hs-CRP levels also correlated with HMOD-HRC in the whole cohort and in the Ow and Ob subpopulations. These findings highlight the need for early intervention targeting ideal CVHMs among hypertensive individuals with an Ow/Ob phenotype in order to attenuate the inflammatory state and prevent cardiovascular disease.

## 1. Introduction

Hypertension in the general population is a growing global health concern. It is related to a higher risk of hypertension-mediated organ damage (HMOD) (i.e., stroke, left ventricular hypertrophy, abnormal ankle–brachial index, and chronic kidney disease) and hypertension-related comorbidities (HRC), such as overt cardiovascular disease (CVD), in addition to mortality, even in patients whose blood pressure is well controlled [1,2,3]. An increased risk for hypertension is plainly linked to overweight/obesity (Ow/Ob), showing that the two conditions are closely related because the cascade of Ow/Ob-induced pathophysiologic adaptations creates a clear path to hypertension [4]. As a consequence, the Ow/Ob-hypertension phenotype is highly prevalent, increasing rapidly worldwide over the last three decades, with its deleterious health implications. Likewise, an abnormal nutritional status of patients with the Ow/Ob-hypertension phenotype is associated with a higher rate of life-threatening complications and longer length of hospitalization, with the corresponding burden on the healthcare system [5]. In addition, sustained inflammation is thought to be a strong risk factor for the development of multiple diseases, including CVD and metabolic disturbances [6]. Indeed, obesity and hypertension have been associated with chronic inflammation in both obese and hypertensive patients regardless of age, ethnicity characteristics, and sex [7,8,9]. Obesity predisposes to a low-grade pro-inflammatory state via increased inflammatory mediators IL-6 and TNF-α, which in turn triggers hepatocyte expression and release of serum high-sensitive C-reactive protein (hs-CRP). This low-grade inflammatory state is followed by vascular and endothelial dysfunction from decreased nitric oxide and elevated reactive oxygen species, leading to oxidative stress and predisposing to hypertension and atheromatous-related cardiovascular events [4].

The American Heart Association (AHA) has developed a metric, termed Life’s Simple 7 (LS7), the aim of which is to define and promote ideal cardiovascular health metrics (CVHM) [10]. The LS7 includes four modifiable health behaviors (body mass index [BMI] < 25 kg/m^2^, physical activity, a healthy diet, and nonsmoking) and three modifiable biological factors (untreated total cholesterol <200 mg/dL, untreated blood pressure <120/80 mmHg, and untreated fasting blood glucose <100 mg/dL). An inverse relationship between CVHM score and HMOD-HRC and diabetes-related life-threatening complications has been documented in both hypertensive and diabetic populations [11,12,13]. Moreover, ideal CVHM have been related to decreased hs-CRP levels in hypertensive patients [14,15].

A priori, Ow/Ob and hypertension are key unfavorable health metrics. Both obese and hypertensive patients share many of the LS7 risk factors, but the relationship between having an ideal LS7 risk factor profile in patients with Ow/Ob-hypertension phenotype plus low-grade inflammation and their medical complications has not been thoroughly assessed. Therefore, identifying patients with these phenotypes and more aggressive treatment focused on weight loss and blood pressure control could improve adverse outcomes in this particular population.

We hypothesized that a poorer cardiovascular health status plus a low-grade inflammatory state is associated with a greater risk of Ow/Ob hypertension-related phenotype adverse outcomes. Thus, we aimed to determine the association between the CVHM score and the presence of low-grade inflammation and Ow/Ob hypertension-associated phenotype morbidities and mortality in patients with hypertension belonging to an urban primary care center.

## 2. Materials and Methods

### 2.1. Study Population

The study included 243 consecutive adult Caucasian hypertensive patients attended in a Spanish urban healthcare center (La Cuesta, La Laguna, Tenerife, Canary Islands) between September 2018 and September 2019 and who provided informed consent.

We enrolled both prevalent hypertensive patients from our primary care center (*n* = 233) as well as prevalent hypertensive patients referred from other care centers (*n* = 10). Prevalent patients were those patients who already had hypertension at the time of recruitment. All patients had a diagnosis of primary hypertension and the median follow-up was 156.5 months (interquartile range 84–204 months).

**Figure 1:**
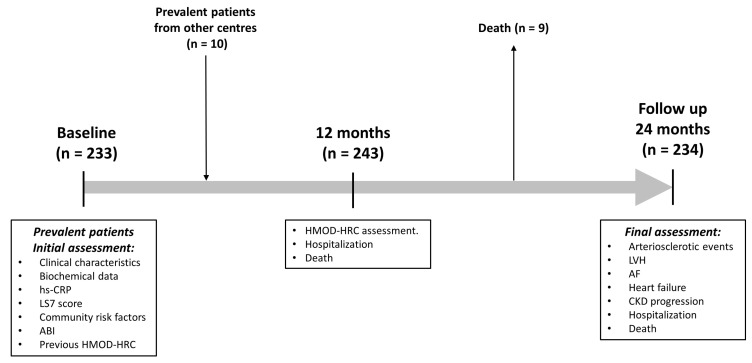
Study flowchart. Abbreviations: ABI, ankle–brachial index; AF, atrial fibrillation; CKD, chronic kidney disease; LVH, left ventricular hypertrophy; LS7, Life’s Simple 7; HMOD-HRC, hypertension-mediated organ damage and hypertension-related comorbidities; hs-CRP, high-sensitivity C-reactive protein.

Follow-up was censored up to the end of the observation period (2 years after baseline assessment) or at the time of death. All surviving patients were therefore followed for at least 24 months from inclusion. Approval for the study protocol was granted by the institutional review board of University Regional Hospital of Malaga and it complied with the Declaration of Helsinki. Figure 1 shows the study flowchart.

### 2.2. Data Collection

A detailed medical history was collected from all patients using a standardized questionnaire, as previously reported [13,16], including socioeconomic and demographic data, marital status, average family income, education level, nocturnal work, physical activity, quality of diet, smoking status, salt intake, alcohol consumption, and active treatment such as anti-hypertensive drugs, lipid-lowering agents, hypoglycemic agents, and family history of hypertension, diabetes, and CVD. Evaluations were also performed for renal function assessed by MDRD equations and chronic kidney disease (CKD), defined as eGFR < 60 mL/min/1.73 m^2^ and/or an albumin–creatinine ratio > 30 mg/g measured at a minimum of two different times during a period of 3 or more months, regardless of the cause.

Additionally, we assessed at baseline pre-existing HMOD-HRC according to standard definitions from clinical practice guidelines [3]. We also ascertained HMOD-HRC during the follow-up period of two years. HMOD included stroke, left ventricular hypertrophy (LVH), peripheral artery disease (PAD), and CKD. HRC included arteriosclerotic events (coronary artery disease [CAD], stroke and symptomatic PAD), heart failure, atrial fibrillation, and CKD or its progression. CAD was defined as myocardial infarction or coronary artery revascularization using standard criteria [17]. Atrial fibrillation and LVH were determined by electrocardiography. Stroke was diagnosed as a persistent central neurologic deficit lasting 24 h and unexplained by other reasons. Heart failure was diagnosed by clinical criteria and dilated ventricle or poor left ventricular function. PAD was defined as having an abnormal ankle–brachial index, evaluated by an improved automated oscillometric device (MESI, ABPI MDR system, Ljubljana, Slovenia) [18], or history of intermittent claudication, leg revascularization, or amputation. CKD progression was defined as a 50% decrease in eGFR from baseline or occurrence of end-stage renal disease. Lastly, we assessed mortality from any cause during follow-up.

### 2.3. Determination of Serum hs-CRP Levels

Serum hs-CRP levels were determined by a commercial particle-enhanced immunoturbidimetric method (Tina-quant CRP4, Roche Cobas 6000 c 501, Mannheim, Germany) with a detection limit of 0.3 mg/L and extended measuring range 0.6–350 mg/L, according to the manufacturer.

### 2.4. Cardiovascular Health Metrics

Seven ideal CVHMs were used from the AHA LS7 definition (Table S1) [10]. Ideal CVH behaviors included never smoking or quitting smoking more than 12 months previously, BMI < 25 kg/m^2^, physical exercise at least 150 min/week of moderate intensity or 75 min/week of vigorous intensity and ideal healthy diet, which was defined as meeting four or five of the following criteria: fish consumption ≥ 2 serving/week, fruit/vegetables ≥ 4.5 cups/day, sodium intake < 1500 mg/day, sugar < 450 kcal/week and fiber–carbohydrate ratio > 0.5. Ideal CVH factors included untreated total serum cholesterol < 200 mg/dL, untreated systolic blood pressure < 129 mmHg and diastolic blood pressure < 80 mmHg, and untreated serum fasting glucose < 100 mg/dL. Individuals were grouped according to whether they had 0–1, 2–3 or 4–5 ideal CVHMs. As all individuals had hypertension, no patient had an ideal LS7 blood pressure category, and only three patients had five ideal CVHMs.

### 2.5. Study Outcomes

The main study outcome was the presence of preexisting or incident HMOD-HRC during follow-up. Additionally, we assessed data on mortality at the end of follow-up. Thus, an additional composite endpoint was generated that included global HMOD (preexisting and incident) and all-cause mortality.

### 2.6. Statistical Analysis

A descriptive analysis of the results was performed, expressing the quantitative variables as the mean ± standard deviation for parametric data. Skewed continuous variables were natural log transformed before the analysis. Categorical variables were expressed as numbers and percentages. Comparisons of quantitative variables were made by Student’s t-test or the Mann–Whitney U test as appropriate. Categorical variables were compared by the chi-square test or Fisher’s exact test as appropriate. Patients were stratified into three groups according to BMI: (1) Normal (BMI < 25 kg/m^2^); (2) Overweight (BMI 25–29.9 kg/m^2^); and (3) Obese (BMI ≥ 30 kg/m^2^). Additionally, the patients were also divided into three groups by log hs-CRP-level tertiles. Baseline characteristics were compared across the BMI groups and log hs-CRP groups using one-way ANOVA for continuous variables and the chi-square test for categorical variables. Correlations between clinical parameters were performed by univariate regression analysis. We also performed multivariate linear regression analysis of factors associated with hs-CRP levels. Multiple binary logistic regression models were undertaken to assess the association between cumulative scores and the endpoints after adjustment for confounders. Additionally, we also examined the relationship between log hs-CRP levels and composite endpoints. The Hosmer–Lemeshow goodness of fit was the main criterion for selection of the final models. Statistical analysis was performed with SPSS Statistics V26.0 for Windows (IBM Corp., Armonk, NY, USA), and the significance was set at *p* < 0.05.

## 3. Results

Of the 243 participants, 26 (10.7%) had BMI < 25 kg/m^2^, 95 (31.1%) were overweight (BMI 25–29.9 kg/m^2^), and 122 (50.2%) were obese (BMI ≥ 30 kg/m^2^). No patients were underweight (BMI < 18.5 kg/m^2^). Overall, the mean age was 68.6 ± 13 years. Table 1 displays socio-demographic and clinical characteristics, including community risk factors and CVHMs, according to BMI stratification of the entire cohort. Higher hs-CRP levels were observed as BMI increased in overweight and obese patients. As expected, the obese patient group contained a numerically higher proportion of diabetics as well as having significantly higher HbA1c levels and lower HDL cholesterol levels compared with the rest. Lastly, obese and overweight patients presented lower CVHM scores than individuals with a normal BMI.

### 3.1. Baseline Characteristics According to the LS7 Factors

**Figure 2:**
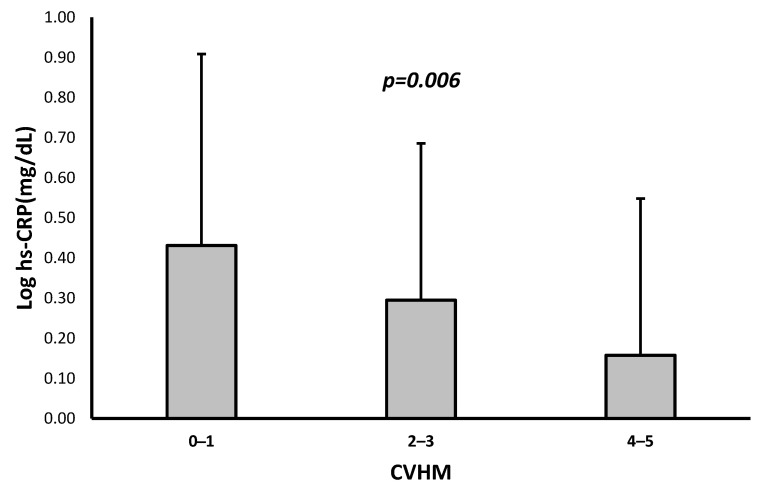
Relationship between log hs-CRP and CVHM score.

In comparison to patients with a healthier score of 4–5, those with a score of 0–1 or 2–3 had a significantly higher proportion of diabetes and higher fasting glucose, HbA1c, and log triglyceride levels as well as a higher BMI (Table 2). Likewise, a higher log albumin–creatinine ratio was also observed in patients with a score of 0–1 compared with the rest. Finally, a higher proportion of prevalent CVD and HMOD-HRC was observed in individuals with a score of 0–1 or 2–3 compared with patients with a healthier score (Table 2). Interestingly, significantly lower log hs-CRP levels were observed as the number of ideal CVHM scores increased (Figure 2).

### 3.2. Relationship between BMI and hs-CRP Levels

**Figure 3:**
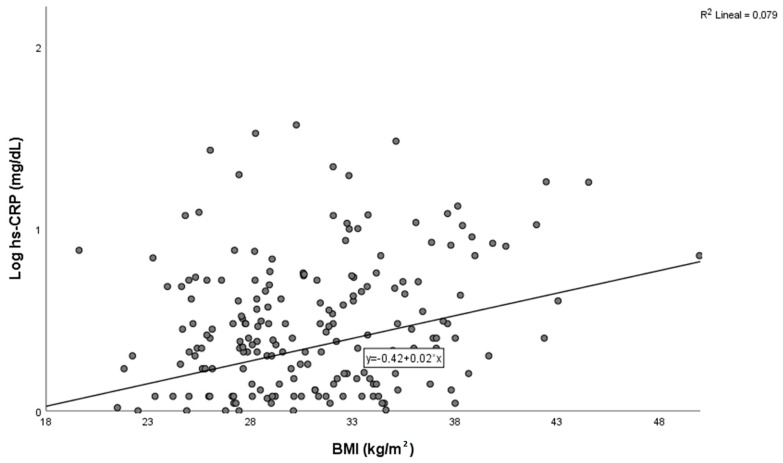
Correlation between Log hs-CRP and Body Mass Index (BMI).

Table 3 shows clinical characteristics according to log hs-CRP tertiles of the whole population. Compared to patients with the lowest log hs-CRP tertile, those with a higher log hs-CRP tertile (second and third) had a significant increase in BMI. Likewise, an increased albumin–creatinine ratio was also observed in patients with the highest log hs-CRP tertile compared with the rest. Lastly, compared to patients with the lowest log hs-CRP tertile, those with a higher log hs-CRP had a significantly higher proportion of CKD. A significant correlation between BMI and hs-CRP was also seen in the whole population (Figure 3). Additionally, linear regression analysis again showed a significant correlation between BMI and hs-CRP adjusted for confounder variables such as age, gender, albumin–creatinine ratio and the presence of CKD (Table 4).

### 3.3. Effect of Cumulative CVHMs and Log hs-CRP Levels on Endpoints

There were 264 cases of HMOD-HRC in 243 patients, with 225 recorded in the initial analysis and 39 during follow-up. In total, 58 patients presented more than one HMOD-HRC. In addition, 9 deaths were recorded: 6 from CVD, 2 from cancer, and 1 from infectious complications. As expected, patients with HMOD-HRC were older, had had hypertension for a longer time, with more having diabetes, in addition to a lower GFR and higher albumin–creatinine ratio (Table S2). Moreover, a higher proportion of ideal CVHM scores was also found in patients without HMOD-HRC. Finally, a trend toward higher log hs-CRP levels was seen in patients with HMOD-HRC and all deaths occurred in those with these complications.

Multivariate binary logistic regression analysis adjusted for confounders (Table 5) showed the risk of HMOD-HRC increased significantly as the number of ideal CVHM scores decreased, using individuals with 4 to 5 ideal CVHM scores as the reference in the entire cohort. Interestingly, log hs-CRP levels also correlated with the dependent variable (HMOD-HRC) regardless of CVHM score. Similarly, when this analysis was performed in both overweight and obese patients, again log hs-CRP correlated with the presence of HOMD-HRC but not the number of ideal CVHM scores.

## 4. Discussion

### 4.1. Summary of Findings

The main findings of this longitudinal cohort study performed in an urban hypertensive population are: (1) a close relation between BMI and hs-PCR levels, and unsurprisingly, this correlation was greatest in the obese group; (2) an increasing number of ideal CVHM scores was associated with decreasing hs-CRP levels; and (3) overall CVHM score and hs-CRP are independently associated with HMOD-HRC, particularly marked by hs-CRP in overweight and obese individuals. Globally, this suggests that an Ow/Ob hypertension phenotype is associated with low-grade inflammation and development of HMOD-HRC. This is one of only a few prospective studies to assess the linking mechanism between obesity-associated inflammation and hypertension with poor CVH and unfavorable outcomes in this particular population. Thus, implementing strategies to improve CVH may be an effective real-life approach to the prevention of inflammatory dysregulation in at-risk populations, as are hypertensive patients.

### 4.2. Comparison with Previous Studies and Risk Factors, Including hs-CRP Levels

Hypertension is one of the principal risk factors for cardiovascular disease [1], and low-grade inflammation, defined as a condition with chronically elevated concentrations of inflammatory parameters such as serum hs-CRP levels, is believed to play a fundamental role in CVD in several clinical settings as well as in population-based studies [19,20,21,22]. Indeed, this low-grade inflammatory state is very common in multiple chronic conditions highly prevalent worldwide, particularly in Western populations with hypertension and an Ow/Ob phenotype [23,24]. Increased CRP levels precede and predict the development of hypertension and type 2 diabetes, suggesting that CRP itself may be pathophysiologically important [25,26]. Likewise, many unfavorable health behaviors and biological factors favoring the development of hypertension, comprised in the CVHMs, have been identified [3]. A high prevalence of Ow/Ob was seen in our hypertensive population. We also observed a significant reduction in hs-CRP levels as the ideal CVHM score increased. In consonance with our findings, previous studies have demonstrated that the number of ideal CVHM is negatively correlated with hs-CRP levels in hypertensive patients leading to atheromatous-associated cardiovascular events [14,15]. It is plausible to think, therefore, that the cardiovascular protective effects of ideal CVHM could result from the reduction in serum CRP levels through intricate mechanisms such as reduction in BMI or fasting blood glucose, among others. Obesity and components of metabolic syndrome may induce systemic inflammation leading to endothelial dysfunction by affecting nitric oxide synthesis or degradation, which may increase hs-CRP, especially when obesity and hypertension concur [27,28]. Indeed, adipocytes express and secret TNF-alpha and adipose tissues are able to increase the secretion of IL-6, which is involved in the pathogenesis of the production of CRP in the liver [29,30]. Moreover, weight loss through diet or after gastric bypass surgery is related to a reduction in serum IL-6, TNF-alpha, and CRP levels [31,32]. In this line, a systematic review and meta-analysis of cross-sectional studies relating CRP and obesity demonstrated a strong association between CRP and BMI [7]. As a matter of fact, in our hypertensive population hs-CRP levels were found to increase as BMI increased, and BMI was associated with hs-CRP in the multivariate linear regression analysis after adjusting for confounders. Consequently, a greater prevalence of CVD and CKD as well as a higher albumin–creatinine ratio were observed in our patients with the highest log hs-CRP tertile. Thus, although mechanisms of the inverse relationship between ideal CVH and CRP still remain unclear, these arguments may partly explain the negative association between CVHM and hs-CRP levels found in our study.

Overweight and obesity are two of the most significant risk factors that increase the risk of hypertension [19], suggesting that these two conditions are intimately related because the Ow/Ob phenotype-induced pathophysiologic adaptations lead to a clear pathway to hypertension. In addition, serum CRP levels have been reported to increase with augmenting BMI [28,33,34], as observed in our study. It is, thus, unsurprising that increased CRP levels could be associated with an increased risk of HMOD-HRC in a hypertensive population where a myriad of risk factors concur, as seen in the current study. In fact, hs-CRP was significantly associated with overall unfavorable outcomes (HMOD-HRC and mortality) in our hypertensive patients independently of other known risk factors, including a poor CVH score. Importantly, this significant association was again observed in the overweight and obese subpopulations after adjustment for confounders. Taken together, therefore, a linking mechanism between Ow/Ob-associated CRP levels and HMOD-HRC in hypertensive patients seems likely. The fact that reduction in inflammation, evaluated by CRP levels, has been associated with a reduction in HMOD with uric-acid-lowering therapy in patients with essential hypertension [35] supports this hypothesis. Further large prospective intervention studies are needed to demonstrate that advocating ideal CVHM may decrease hs-CRP, leading to prevention of hypertension-related complications.

### 4.3. Implications

Given the association between increased CRP levels and the absence of an ideal CVH behavior and biological factors that make up the CVHM score, including in the hypertensive population with an Ow/Ob phenotype, efforts should be made by clinicians to identify and treat modifiable behavior and biological risk factors (e.g., proper control of blood pressure and lipid and glucose levels, reduction in BMI, stopping smoking, healthy diet, and physical exercise) to prevent hypertension-related complications through attenuation of the inflammatory state in these patients. Finally, because the relationship between a reduction in CRP levels and cardiovascular protection has not been fully demonstrated in clinical trials, we suggest that CRP levels should not be used to guide therapy, but to identify subgroups of hypertensive patients with an Ow/Ob phenotype at a high CV risk.

### 4.4. Limitations and Strengths

This study has several limitations. The sample of hypertensive patients was quite small and further studies with larger sample sizes are required in this particular population. Our patients were generally old, had multiple comorbidities and came from a Caucasian urban population, which could have led to selection bias. In fact, rural populations or those from non-Caucasian races are unique in comparison to metropolitan populations and Caucasians. Thus, application of these results to other areas should be interpreted cautiously. We only determined hs-CRP as the inflammatory parameter and no other inflammatory markers, such as TNF-alpha or IL-6, were assessed. Furthermore, no data were collected on adherence to anti-hypertensive medication, which could also underrate the impact on the outcome of CVH metrics and hs-CRP. Additionally, as the data collected were partly self-reported, social desirability bias was also probable. Lastly, the study results cannot themselves clarify whether changes in health measures can in fact lower hs-CRP and HMOD-HRC and mortality. Indeed, although therapeutic and preventive strategies were applied during follow-up in those patients with lower ideal CVH metrics to optimize their CVH, we did not evaluate whether changes in CVHMs really decreased hs-CRP levels and unfavorable outcomes.

Our study also has some strengths. We not only followed strictly the recommendations of the LS7 to achieve ideal CVHMs, but we also recorded other community risk factors such as nocturnal work, education level, alcohol consumption, or family income, which could influence CVH metrics negatively. Secondly, collection of baseline data was comprehensive and thorough, with the use of a standardized questionnaire, and the current study had no missing data.

## 5. Conclusions

This study demonstrated that increasing numbers of ideal CVHMs were associated with decreasing hs-CRP levels and hypertensive patients with both an Ow/Ob phenotype and higher hs-CRP levels may have unfavorable outcomes through an inflammatory mechanism. Thus, our findings emphasize the importance of early intervention targeting both lifestyle behaviors and biological risk factors to achieve ideal CVHMs among hypertensive individuals with an Ow/Ob phenotype in order to attenuate the inflammatory state and prevent CVD.

## Figures and Tables

**Table 1:** Baseline socio-demographic and clinical characteristics according to BMI stratification.

Characteristics *	Overall (*n* = 243)	BMI < 25 (*n* = 26)	BMI 25–29.9 (*n* = 95)	BMI ≥ 30 (*n* = 122)	*p*-Value
Age (years)	68.5 ± 13	67.7 ± 13.7	68.3 ± 13.1	69.0 ± 12.9	0.858
Sex (male, %)	46.1	34.6	53.7	42.6	0.124
Duration of hypertension (months)	156 ± 109	131.6 ± 82.9	153.5 ± 109.5	164.2 ± 114.5	0.362
BMI, (kg/m^2^)	30.5 ± 5	23.3 ± 1.5	27.6 ± 1.3	34.5 ± 4.9	<0.001
Waist–height ratio	0.63 ± 0.07	0.54 ± 0.05	0.60 ± 0.05	0.69 ± 0.07	<0.001
SBP, (mmHg)	135.3 ± 17	129.3 ± 14.2	134.8 ± 17.0	137.0 ± 17.2	0.108
DBP, (mmHg)	80 ± 9.6	76.6 ± 8.0	79.9 ± 9.8	80.7 ± 9.7	0.139
Fasting glucose (mg/dL)	110 ± 36	104.7 ± 38.2	105.7 ± 28.4	114.4 ± 41.6	0.164
Total cholesterol (mg/dL)	187 ± 37	196.8 ± 31.5	190.2 ± 39.9	182.4 ± 36.8	0.119
HDL cholesterol (mg/dL)	50.7 ± 14	59.9 ± 14.8	50.7 ± 14.2	48.7 ± 12.8	0.001
LDL cholesterol (mg/dL)	106 ± 53	109.6 ± 27.6	109.4 ± 37.5	103.1 ± 34.4	0.371
Log triglycerides (mg/dL)	2.1 ± 0.2	2.1 ± 0.2	2.1 ± 0.2	2.1 ± 0.2	0.355
Diabetes (%)	41.6	34.6	33.7	49.2	0.053
Family history of CVD ^ (%)	60	50	61.1	61.5	0.538
Family history of hypertension (%)	79.8	76.9	76.8	82.8	0.515
Family history of diabetes (%)	56	42.3	58.9	56.6	0.312
Family history of cancer (%)	60.5	69.2	64.2	55.7	0.282
Education level (%)					0.774
Primary school or below	71.6	65.4	71.6	73.0	
Middle school	21.8	23.1	21.1	22.1	
High school or university	6.6	11.5	7.4	4.9	
Marital status (%)					0.043
Married or cohabiting	61.3	38.5	68.4	60.7	
Single or religious	5.3	3.8	6.3	4.9	
Divorced or widowed	33.3	57.7	25.3	34.4	
Alcohol consumption **(%)					0.929
Low	76	80.8	74.7	76.2	
Moderate	19	15.4	21.1	18.0	
High	5	3.8	4.2	5.7	
Low income ^^ (%)	83.5	80.8	82.1	85.2	0.735
Nocturnal work (%)	6.2	7.7	6.3	5.7	0.929
eGFR (ml/min/1.73 m^2^)	78.3 ± 21.5	78.1 ± 13.1	79.3 ± 20.5	77.6 ± 23.8	0.854
Log albumin–creatinine (mg/g)	1.3 ± 0.7	1.3 ± 0.7	1.3 ± 0.6	1.3 ± 0.8	0.856
HbA1C (%)	6 ± 1	6.0 ± 0.9	5.8 ± 0.8	6.2 ± 1.2	0.023
Log C-reactive protein (mg/dL)	0.33 ± 0.4	0.15 ± 0.4	0.28 ± 0.4	0.42 ± 0.4	0.003
ABI	1.17 ± 0.2	1.18 ± 0.1	1.19 ± 002	0.16 ± 0.2	0.575
Antihypertensive medication (%)					
ACEI/ARA II	92.2	92.3	90.4	94.3	0.566
Calcium antagonists	30	15.4	25.3	36.9	0.041
Beta-blockers	19.8	15.4	20.2	20.5	0.833
Vasodilators	4	0	3.2	5.7	0.341
Diuretics	50.6	42.3	46.8	55.7	0.281
Lipid-lowering medication (%)	55	42.3	54.7	58.2	0.333
Left ventricular hypertrophy (%)	22	15.4	24.2	21.3	0.616
CVD (%)	42	34.6	41.1	44.3	0.646
CKD (%)	16.9	7.7	15.8	19.7	0.313
Hospitalization (%)	26.3	26.9	28.4	24.6	0.815
Death (%)	3.7	0	3.2	4.9	0.453
HMOD-HRC (%)	50.2	46.2	45.3	54.9	0.336
Cardiovascular health metric scores %					<0.001
0–1	41.2	0.0	36.8	53.3	
2–3	48.1	57.7	52.6	42.6	
4–5	10.7	42.3	10.5	4.1	

Abbreviations: ABI, ankle–brachial index; ACEI/ARA II, angiotensin-converting enzyme inhibitors or angiotensin receptor antagonists; BMI, body mass index; CKD, chronic kidney disease; CVD, cardiovascular disease; DBP, diastolic blood pressure; eGFR, estimated glomerular filtration rate; HMOD-HRC, hypertension-mediated organ damage and hypertension-related comorbidities; SBP, systolic blood pressure. *: mean (SD) unless otherwise stated. ** low: <10 g/d (men), <5 g/d (women); moderate: 10–50 g/d (men), 5–10 g/d (women); high: ≥50 g/d (men), ≥10 g/d (women). ^ CVD, cardiovascular disease, including myocardial infarction, heart failure, cardiac arrhythmia, stroke, and peripheral vascular disease. ^^ low income: < EUR 25,000/y.

**Table 2:** Clinical and socio-demographic characteristics according to the CVHM score.

Characteristics *	Overall (*n* = 243)	CVHM 0–1 (*n* = 100)	CVHM 2–3 (*n* = 117)	CVHM 4–5 (*n* = 26)	*p*-Value
Age (years)	68.5 ± 13	69.4 ± 12.7	68.7 ± 12.8	65.1 ± 15.4	0.321
Sex (Male, %)	46.1	47.0	44.4	50.0	0.852
Duration of hypertension (months)	156 ± 109	168.6 ± 121.9	153.3 ± 103.2	124.0 ± 79.5	0.165
BMI, (kg/m^2^)	30.5 ± 5	32.1 ± 4.4	30.1 ± 5.1	27.1 ± 4.2	<0.001
Waist–height ratio	0.63 ± 0.07	0.66 ± 0.8	0.63 ± 0.07	0.59 ± 0.07	<0.001
SBP, (mmHg)	135.3 ± 17	137.7 ± 18	133.9 ± 16.8	132.6 ± 14.8	0.185
DBP, (mmHg)	80 ± 9.6	79.8 ± 10.2	79.7 ± 9.5	81.2 ± 8.0	0.769
Fasting glucose (mg/dL)	110 ± 36	124.9 ± 44.7	101.5 ± 26.9	90.5 ± 13.9	<0.001
Total cholesterol (mg/dL)	187 ± 37	184.5 ± 39.3	190.3 ± 39.3	182.2 ± 20.3	0.421
HDL cholesterol (mg/dL)	50.7 ± 14	47.4 ± 13.6	52.7 ± 14.1	54.1 ± 12.3	0.009
LDL cholesterol (mg/dL)	106 ± 53	103.2 ± 36.6	108.8 ± 36.0	106.5 ± 22.3	0.511
Log triglycerides (mg/dL)	2.1 ± 0.2	2.2 ± 0.2	2.1 ± 0.2	2.0 ± 0.2	<0.001
Diabetes (%)	41.6	64	28.2	15.4	<0.001
Family history of CVD ^ (%)	60	59	61.5	57.7	0.898
Family history of hypertension (%)	79.8	78	82.1	76.9	0.704
Family history of diabetes (%)	56	62.0	52.1	50.0	0.280
Family history of cancer (%)	60.5	61	59.8	61.5	0.978
Education level (%)					0.814
Primary school or below	71.6	74	70.9	65.4	
Middle school	21.8	20	23.1	23.1	
High school or university	6.6	6	6	11.5	
Marital status (%)					0.577
Married or cohabiting	61.3	64	62.4	46.2	
Single or religious	5.3	5	5.1	7.7	
Divorced or widowed	33.3	31	32.5	46.2	
Alcohol consumption ** (%)					0.149
Low	76	77	73.5	84.6	
Moderate	19	15	23.9	11.5	
High	5	8	2.6	3.8	
Low income ^^ (%)	83.5	86	83.8	73.1	0.358
Nocturnal work (%)	6.2	5	6.8	7.7	0.806
eGFR (ml/min/1.73 m^2^)	78.3 ± 21.5	76.3 ± 23.6	79.0 ± 20.5	82.9 ± 16.6	0.345
Log hs-C-reactive protein (mg/dL)	0.34 ± 0.44	0.43 ± 0.48	0.30 ± 0.39	0.16 ± 0.39	0.006
Log albumin–creatinine (mg/g)	1.3 ± 0.7	1.5 ± 0.7	1.1 ± 0.6	1.2 ± 0.7	0.025
HbA1C (%)	6 ± 1	6.5 ± 1.3	5.8 ± 0.7	5.7 ± 0.7	<0.001
ABI	1.17 ± 0.2	1.16 ± 0.2	1.17 ± 0.2	1.21 ± 0.1	0.395
Antihypertensive medication (%)					
ACEI/ARA II	92.2	92.9	93.2	88.5	0.699
Calcium antagonists	30	34	29.1	19.2	0.325
Beta-blockers	19.8	19.2	23.9	3.8	0.066
Vasodilators	4	4	5.1	0	0.491
Diuretics	50.6	5.5	53.8	30.8	0.094
Lipid-lowering medication (%)	55	69	53.8	7.7	<0.001
Left ventricular hypertrophy (%)	22	25	21.4	11.5	0.330
CVD (%)	42	43	46.2	19.2	0.041
CKD (%)	16.9	21	15.4	7.7	0.227
Hospitalization (%)	26.3	31	24.8	15.4	0.238
Death (%)	3.7	6	2.6	0	0.234
HMOD-HRC (%)	50.2	54	53	23.1	0.014

Abbreviations: ABI, ankle–brachial index; ACEI/ARA II, angiotensin-converting enzyme inhibitors or angiotensin receptor antagonists; BMI, body mass index; CKD, chronic kidney disease; CVD, cardiovascular disease; DBP, diastolic blood pressure; eGFR, estimated glomerular filtration rate; HMOD-HRC, hypertension-mediated organ damage and hypertension-related comorbidities; SBP, systolic blood pressure. *: mean (SD) unless otherwise stated. ** low: <10 g/d (men), <5 g/d (women); moderate: 10–50 g/d (men), 5–10 g/d (women); high: ≥50 g/d (men) ≥10 g/d (women). ^ CVD, cardiovascular disease, including myocardial infarction, heart failure, cardiac arrhythmia, stroke, and peripheral vascular disease. ^^ low income: < EUR 25,000/y.

**Table 3:** Clinical characteristics according log hs-CRP tertiles.

	Log hs-CRP (mg/dL)				
Characteristics *	Overall (*n* = 243)	Tertile 1 (<0.08, *n* = 82)	Tertile 2 (0.08–0.45, *n* = 81)	Tertile 3 (>0.45, *n* = 80)	*p*-Value
Age (years)	68.5 ± 13	69.5 ± 13.0	67.2 ± 13.3	39.2 ± 12.9	0.498
Sex (Male, %)	45.8	50	48.1	39.0	0.331
Duration of hypertension (months)	156 ± 109	125.6 ± 111.5	158.6 ± 109.7	158.3 ± 110.4	0.928
BMI, (kg/m^2^)	30.5 ± 5	28.8 ± 4.4	30.7 ± 4.3	32.2 ± 5.5	<0.001
Waist–height ratio	0.63 ± 0.07	0.61 ± 0.07	0.65 ± 0.07	0.66 ± 0.08	<0.001
SBP, (mmHg)	135.3 ± 17	136.3 ± 16.6	134.3 ± 17.9	135.3 ± 17.5	0.773
DBP, (mmHg)	80 ± 9.6	80.1 ± 9	80.4 ± 10.4	79.4 ± 9.5	0.821
Fasting glucose (mg/dL)	110 ± 36	106.8 ± 31.9	107.3 ± 29.8	115.4 ± 46.8	0.257
Total cholesterol (mg/dL)	187 ± 37	187.5 ± 37.8	184.7 ± 36.7	188.7 ± 37.9	0.788
HDL cholesterol (mg/dL)	50.7 ± 14	53.3 ± 14.7	49.1 ± 12.4	49.8 ± 14.5	0.123
LDL cholesterol (mg/dL)	106 ± 53	107.2 ± 36.4	105.9 ± 36	106.3 ± 33.2	0.971
Log triglycerides (mg/dL)	2.1 ± 0.2	2.1 ± 0.21	2.1 ± 0.21	2.2 ± 0.20	0.043
Diabetes (%)	41.6	34.1	42.0	49.4	0.151
Family history of CVD ^ (%)	60	59.8	65.4	55.8	0.463
Family history of hypertension (%)	79.8	75.6	75.2	79.2	0.305
Family history of diabetes (%)	56	52.4	60.5	54.5	0.563
Family history of cancer (%)	60.5	62.2	55.6	64.9	0.460
Education level (%)					0.941
Primary school or below	71.6	70.7	71.6	72.7	
Middle school	21.8	20.7	22.2	22.1	
High school or university	6.6	8.5	6.2	5.2	
Marital status (%)					0.705
Married or cohabiting	61.3	56.1	61.7	66.2	
Single or religious	5.3	4.9	6.2	5.2	
Divorced or widowed	33.3	39.0	32.1	28.6	
Alcohol consumption ** (%)					0.287
Low	76	80.5	74.1	74.0	
Moderate	19	17.1	22.2	16.9	
High	5	2.4	3.7	9.1	
Low income ^^ (%)	83.5	73.2	85.2	92.2	0.006
Nocturnal work (%)	6.2	9.8	4.9	3.9	0.261
eGFR (mL/min/1.73 m^2^)	78.3 ± 21.5	80.0 ± 17.9	75.5 ± 20.5	79.7 ± 24.3	0.317
Log albumin–creatinine (mg/g)	1.3 ± 0.7	1.1 ± 0.5	1.2 ± 0.6	1.7 ± 0.7	0.004
HbA1C (%)	6 ± 1	5.9 ± 0.9	6.0 ± 1.0	6.2 ± 1.2	0.410
ABI	1.17 ± 0.2	1.19 ± 0.13	1.16 ± 0.22	1.17 ± 0.17	0.672
Antihypertensive medication (%)					
ACEI/ARA II	92.2	92.7	91.4	93.5	0874
Calcium antagonists	30	31.7	25.9	32.5	0.613
Beta-blockers	19.8	15.9	22.2	20.8	0.562
Vasodilators	4	3.7	2.5	5.2	0.665
Diuretics	50.6	43.9	44.4	64.9	0.011
Lipid-lowering medication (%)	55	58.5	50.6	54.5	0.597
Left ventricular hypertrophy (%)	22	19.5	24.7	20.8	0.706
CVD (%)	42	37.8	45.7	41.6	0.595
CKD (%)	16.9	11	25.9	23.4	0.039
Hospitalization (%)	26.3	20.7	25.9	31.2	0.323
Death (%)	3.7	2.4	3.7	3.9	0.855
HMOD-HRC (%)	50.2	47.6	51.9	50.6	0.852
Cardiovascular health metric scores %					0.134
0–1	40.8	34.1	37.0	51.9	
2–3	48.3	51.2	51.9	412.6	
4–5	10.8	14.6	11.1	6.5	

Abbreviations: ABI, ankle–brachial index; ACEI/ARA II, angiotensin-converting enzyme inhibitors or angiotensin receptor antagonists; BMI, body mass index; CKD, chronic kidney disease; CVD, cardiovascular disease; DBP, diastolic blood pressure; eGFR, estimated glomerular filtration rate; HMOD-HRC, hypertension-mediated organ damage and hypertension-related comorbidities; SBP, systolic blood pressure. *: mean (SD) unless otherwise stated. ** low: <10 g/d (men), <5 g/d (women); moderate: 10–50 g/d (men), 5–10 g/d (women); high: ≥50 g/d (men) ≥10 g/d (women). ^ CVD, cardiovascular disease, including myocardial infarction, heart failure, cardiac arrhythmia, stroke, and peripheral vascular disease. ^^ low income: < EUR 25,000/y.

**Table 4:** Multivariate linear regression analysis of factors associated with log hs-CRP.

Variable	Beta	Standardized Beta Coefficient	95% CI	*p*-Value
BMI (Kg/m^2^)	0.035	0.365	0.014–0.056	0.001
Age (years)	−0.001	−0.042	−0.010–0.007	0.722
Sex (Male, %)	−0.083	−0.092	−0.266–0.100	0.368
Log albumin–creatinine (mg/g)	0.109	0.160	−0.064–0.282	0.215
Diabetes (%)	0.056	0.062	−0.163–0.275	0.610
CKD (%)	0.090	0.087	−0.162–0.342	0.479

Abbreviations: BMI, body mass index; CKD, chronic kidney disease.

**Table 5:** Multivariate logistic regression analysis of factors associated with HMOD-HRC in the overweight and all patients and obese subgroups.

Category	OR (95% CI)	*p*-Value
All patients, *n* = 243		
Median Age (≤69 vs. >69 years)	5.753 (3.152–10.501)	<0.001
Sex (M)	2.330 (1.288–4.216)	0.005
CVHM score (%)		
4–5 (ref.)	1	
2–3	4.265 (1.452–12.527)	0.008
0–1	4.348 (1.464–12.912)	0.008
Log hs-CRP (mg/dL)	2.183 (1.202–3.963)	0.010
Overweight (25–29.9 kg/m^2^), *n* = 95		
Median Age (≤69 vs. >69 years)	8.769 (3.178–24.193)	<0.001
Sex (M)	2.456 (0.897–6.725)	0.080
CVHM score (%)		
4–5 (ref.)	1	
2–3	2.692 (0.394–18.403)	0.313
1–2	2.465 (0.343–17.738)	0.370
Log hs-CRP (mg/dL)	4.042 (1.104–14.796)	0.035
Obese (≥30 kg/m^2^), *n* = 122		
Median Age (≤69 vs. >69 years)	6.938 (2.746–17.531)	<0.001
Sex (M)	3.030 (1.203–7.634)	0.019
CVHM score (%)		
4–5 (ref.)	1	
2–3	2.295 (0.298–17.692)	0.425
0–1	3.197 (0.419–24.386)	0.262
Log hs-CRP (mg/dL)	2.615 (1.047–6.532)	0.040

Abbreviations: CVHM, cardiovascular health metric; hs-CRP, high-sensitivity C-reactive protein.

## Data Availability

Data are available on request due to privacy restrictions. The data presented in this study are available on request from the corresponding author. In compliance with Spanish Organic Law 15/1999, the data are not publicly available.

## Supplementary Materials

The following supporting information can be downloaded at: https://www.mdpi.com/article/10.3390/jcdd10070300/s1, Table S1: Definitions for the three category indicators of CVH (poor, intermediate, and ideal), as per American Heart Association specifications. Table S2: Characteristics of patients with and without hypertension-mediated organ damage and hypertension-related comorbidities.
