# Supplementary material for: Association between Cardiovascular Health, C-Reactive Protein, and Comorbidities in Spanish Urban-Dwelling Overweight/Obese Hypertensive Patients
Source: J Cardiovasc Dev Dis. 2023 Jul 13;10(7):300. doi: 10.3390/jcdd10070300 (PMC10380879; doi:10.3390/jcdd10070300)
Supplement: Supplementary file 1 [file jcdd-10-00300-s001.zip › Table S2.docx]

**Table S2.** Characteristics of patients with and without hypertension-mediated organ damage and hypertension-related comorbidities.

| **Characteristics*** | **No HMOD-HRC**  **(n=121)** | **HMOD-HRC**  **(n=122)** | ***P* value** |
| --- | --- | --- | --- |
| **Age, y** | 63.5±12.3 | 73.7±11.7 | <0.001 |
| **Duration of hypertension mo.** | 133±82.9 | 179.7±127 | 0.001 |
| **Body mass index, (kg/m^2^)** | 30.7±5.7 | 30.4±4.1 | 0.627 |
| **Waist–height ratio** | 0.63±0.09 | 0.64±0.07 | 0.225 |
| **Systolic blood pressure, (mmHg)** | 133.2±15.7 | 137.4±18.4 | 0.058 |
| **Diastolic blood pressure (mmHg)** | 81.5±8.9 | 78.4±10.1 | 0.010 |
| **Fasting glucose (mg/dL)** | 105±37.6 | 114.8±35.4 | 0.038 |
| **Total cholesterol (mg/dL)** | 198.8±33.7 | 175.3±38.1 | <0.001 |
| **HDL cholesterol (mg/dL)** | 52±13.5 | 49.3±14.2 | 0.131 |
| **LDL cholesterol (mg/dL)** | 117.7±33.1 | 94.8±33.3 | <0.001 |
| **Log triglycerides (mg/dL)** | 2.1±0.2 | 2.1±0.2 | 0.329 |
| **Diabetes (%)** | 28.9 | 54.1 | <0.001 |
| **Family history of CVD^ (%)** | 55.4 | 64.8 | 0.135 |
| **Family history of hypertension (%)** | 81 | 78.7 | 0.655 |
| **Family history of diabetes (%)** | 51.2 | 60.7 | 0.139 |
| **Family history of cancer (%)** | 63.6 | 57.4 | 0.318 |
| **Education level (%)**  **Primary school or below**  **Middle school**  **High school or university** | 65.3  26.4  8.3 | 77.9  17.2  4.9 | 0.093 |
| **Marital status (%)**  **Married or cohabiting**  **Single or religious**  **Divorced or widowed** | 60.3  7.4  32.2 | 62.3  3.3  34.4 | 0.352 |
| **Alcohol consumption (%)**  **Low**  **Moderate**  **High** | 77.7  15.7  6.6 | 74.6  22.1  3.3 | 0.250 |
| **Low income^^ (%)** | 83.5 | 83.6 | 0.892 |
| **Nocturnal work (%)** | 5.8 | 6.6 | 0.803 |
| **eGFR (ml/min/1.73 m^2^)** | 84.8±15.2 | 71.9±24.8 | <0.001 |
| **Log albuminuria–creatinine (mg/g)** | 1±0.6 | 1.5±0.7 | 0.001 |
| **HbA1C (%)** | 5.9±0.9 | 6.3±1.1 | 0.004 |
| **Log C-reactive protein (mg/dL)** | 0.3±0.5 | 0.4±0.4 | 0.318 |
| **ABI** | 1.2±0.1 | 1.1±0.2 | 0.001 |
| **Antihypertensive medication (%)**  **ACEI/ARA II**  **Calcium antagonists**  **Beta-blockers**  **Vasodilators**  **Diuretics** | 91.7  16.5  8.3  0.8  50.4 | 93.4  43.4  31.4  7.4  51.2 | 0.624  <0.001  <0.001  0.010  0.898 |
| **Lipid-lowering medication (%)** | 41.3 | 68.9 | <0.001 |
| **Left ventricular hypertrophy (%)** | - | 43.4 | <0.001 |
| **CVD^ (%)** | - | 83.6 | <0.001 |
| **CKD (%)** | - | 33.6 | <0.001 |
| **Hospitalization (%)** | 14.9 | 37.7 | <0.001 |
| **Death (%)** | - | 7.4 | 0.002 |
| **Cardiovascular health metrics (%)**  **0–1**  **2**  **3**  **4–5** | 38  28.9  16.5  16.5 | 44.3  32  18.9  4.9 | 0.048 |

Abbreviations: ABI, ankle–brachial index; ACEI/ARA II, angiotensin-converting enzyme inhibitors or angiotensin receptor antagonists; CKD, chronic kidney disease; CVD, cardiovascular disease; eGFR, estimated glomerular filtration rate. HMOD-HRC**,** hypertension-mediated organ damage and/or hypertension-related comorbidities. ^CVD, cardiovascular disease, including myocardial infarction, heart failure, cardiac arrhythmia, stroke, and peripheral vascular disease. ^^low income: <25,000 €/y. *mean (SD) unless otherwise stated
