# Supplementary material for: Association between Cardiovascular Health, C-Reactive Protein, and Comorbidities in Spanish Urban-Dwelling Overweight/Obese Hypertensive Patients
Source: J Cardiovasc Dev Dis. 2023 Jul 13;10(7):300. doi: 10.3390/jcdd10070300 (PMC10380879; doi:10.3390/jcdd10070300)
Supplement: Supplementary file 1 [file jcdd-10-00300-s001.zip › Table S1.docx]

**Table S1**. Definitions for the three category indicators of CVH (poor, intermediate and ideal), as per American Heart Association specifications

| **Cardiovascular health-LS7 indicators** | **Poor** | **Intermediate** | **Ideal** |
| --- | --- | --- | --- |
| Smoking | Current smoker | Former smoker who quit ≤12 mo. ago | Never |
| Total cholesterol (mg/dL) | ≥240 | 200–239 or treated to control | <200 without medication |
| Blood pressure (mmHg) | SBP ≥140 or DBP ≥90 | SBP 120–139 or DBP 80–89 or treated to control | <120/<80 without medication |
| Fasting plasma glucose (mg/dL) | ≥126 | 100–125 or treated to control | <100 without medication |
| Body mass index (kg/m^2^) | ≥30 | 25–29.9 | <25 |
| Physical activity | None | 1–149 min/week moderate intensity or  1–74 vigorous intensity | ≥150 min/week moderate intensity or ≥75 min/week vigorous intensity |
| Healthy diet score* | 0–1 components | 2–3 components | 4–5 components |

CVH, cardiovascular health; SBP, systolic blood pressure; DBP, diastolic blood pressure; LS7, Life’s Simple 7.

*Fruits and vegetables ≥4–5 cups/day; fish ≥2–3 servings/week; fibre-rich whole grains ≥3 servings/day; sodium <1500 mg/day; sugar-sweetened beverages ≤450 kcal/week.
